# Optimal Partitioning for Molecular Phylogenetic Inference and Generic Delimitation in the Cetrarioid Core Group (Parmeliaceae, Ascomycota)

**DOI:** 10.3390/jof12090654

**Published:** 2026-09-01

**Authors:** Chaoyang Liu, Shouyu Guo

**Affiliations:** 1 College of Life Science and Agriforestry, Southwest University of Science and Technology, Mianyang 621010, China; 2 Mycology and Innovative Technology Laboratory, Institute of Microbiology, Chinese Academy of Sciences, Beijing 100101, China

**Keywords:** cetrarioid core group, generic delimitation, large temporal band, lichenized fungi, molecular phylogeny, new combination, new genus, partitioned analysis, secondary structure character, small temporal band

## Abstract

The species diversity of lichenized fungi remains largely underestimated, yet the number of species in the cetrarioid core group of Parmeliaceae has remained stable while generic delimitations have been highly debated. Here, we performed a partitioned phylogenetic analysis incorporating secondary structure characters of three ribosomal loci (ITS, mtSSU, and nuLSU) combined with comprehensive phenotypic traits. Based on the results, we hypothesized that a Large Temporal Band (29.0–35.0 Mya) may serve as a baseline threshold for most generic divergences, corresponding to late-Oligocene global cooling and the primary cladogenesis of cetrarioid core lineages as documented in previous molecular dating studies. Additionally, we suggest a Small Temporal Band (14.0–18.0 Mya) for a subset of recently radiated genera that originated during the Mid-Miocene Climatic Optimum transition (~16 Mya). Two currently accepted genera (*Cetraria* and *Nephromopsis*) are largely supported in their current circumscriptions. Two new genera, *Cetramelanelia* and *Tuckermanoides*, are proposed to accommodate a clade of two species from *Cetrariella* and *Tuckermanopsis platyphylla*, respectively. *Allocetraria* is confirmed to resurrect as a genus separate from *Cetraria*, with *Usnocetraria* and *Vulpicida* treated as its synonyms. *Foveolaria* is reduced to synonymy with *Nephromopsis*. Ten new combinations and one new synonym at the species level are made. A dual-band temporal hypothesis for generic delimitation appears taxonomically reasonable for most macrolichens.

## 1. Introduction

The Parmeliaceae is the most diverse family of lichenized fungi, encompassing over 2700 recognized species [1,2]. Traditional growth-form groups, such as alectorioid, cetrarioid, hypogymnioid, parmelioid, and usneoid, have been described within this family [3,4,5]. Over the past two decades, phylogenetic studies have led to significant taxonomic revisions at various levels [5,6,7,8,9,10]. Cetrarioid lichens, a well-known but taxonomically complex group, are characterized by an erect foliose or subfruticose thallus, a well-developed cortical layer, marginal reproductive structures (apothecia and pycnidia), and associations with green algal photobionts (primarily *Trebouxia* or *Asterochloris* species) [4,11,12,13,14,15]. Ecologically, they are predominantly temperate to boreal in distribution, occurring on diverse substrates including soil, rock, bark, and wood [11,13,14,15,16]. The genus *Cetraria* was initially separated from *Parmelia* based on these traits, with later revisions using anatomy and chemistry to establish many new genera [8,9,10], which reflect the large variation in morphology, cortex anatomy, ascus and conidial characters [15,16].

Although morphological traits have been shown to potentially be polyphyletic, appearing in unrelated lineages across the family Parmeliaceae, numerous molecular studies consistently support a monophyletic core group of cetrarioid lichens. This group included approximately 90 species across 14 genera and is divided into two major clades: “*Cetraria*” and “*Nephromopsis*” [4,8,9,10,17,18,19,20,21]. These findings supported the smaller clades and have redefined the boundaries of genera, but failed to improve the resolution of phylogenetic relationships at the generic level [18,20,21,22]. Consequently, the relationships among several genera remain unclear, with some being identified as non-monophyletic [18] and morphological similarities being observed across unrelated clades [22].

Two approaches are employed to address this challenge. First, increasing taxon sampling in phylogenetic analyses. Simulation studies have shown that adding taxa helps to enhance model parameter estimation and mitigate long-branch attraction, thereby improving phylogenetic accuracy more effectively than increasing the number of characters [23,24]. The inclusion of incomplete data from added taxa can also improve accuracy, particularly when model-based methods such as Bayesian and maximum likelihood methods are used. Second, choosing suitable evolutionary models that account for rate- and pattern-heterogeneity across sites within and between genes is critical [25,26]. Different genes exhibit different evolutionary rates. Even highly conserved genes may contain sites that evolve rapidly, and vice versa. Traditional single-model methods are inadequate for estimating the heterogeneity of substitution rates across sites, which can result in systematic errors, particularly in analyses of large-scale datasets [27,28,29]. Modern approaches highly recommend using advanced partitioning strategies and site-heterogeneous models to mitigate these biases.

Most previous phylogenetic investigations of cetrarioid lichens adopted arbitrary partitioning without referencing rRNA secondary structural features. Moreover, an ongoing taxonomic debate exists regarding generic boundary standards: Divakar et al. (2017) [9] proposed a single uniform Oligocene temporal cutoff for all Parmeliaceae generic separation, while many lichen taxonomists opposed this rigid standard based on phenotypic and palaeoclimatic evidence [5,30]. To fill these research gaps, we constructed an extensive dataset of more samples, curated three standard ribosomal markers (ITS, mtSSU, and nuLSU), and performed a secondary-structure-guided optimal partitioned phylogeny. We further integrated phenotypic, geological, and palaeoclimatic evidence to establish an improved dual temporal-band generic delimitation framework for the cetrarioid core group.

In this study, we expanded the sampling scope as broadly as possible. An extensive taxon set comprising 382 taxa covering 16 genera was constructed. Many specimens from the same species were also incorporated into the analysis when sequence variation was present among them. Three phylogenetic markers associated with the majority of specimens—ITS, mtSSU, and nuLSU—were employed to explore deep phylogenetic relationships. To accommodate site-specific rate variation in rRNA genes, the sequence alignment was partitioned into data subsets based on secondary structure features. The evolutionary rates for each data subset were estimated, and then an optimal partitioning scheme was used for phylogenetic inference.

## 2. Materials and Methods

Specimens Examined: More than 1200 herbarium specimens of cetrarioid lichens deposited at HMAS-L (Lichen Herbarium, Institute of Microbiology, Chinese Academy of Sciences) and KUN (Kunming Institute of Botany, CAS), and some specimens deposited at CANL (visited in 2008) and TNS (visited in 2003), were examined. All examinations were made by the corresponding author (S.Y. Guo) based on standard morphological and chemical criteria [11,12,13,14,15,16,31,32,33,34], and verified against type specimens where available. The important taxonomic characters of morphology, anatomy, and secondary chemistry, as well as data on habitat ecology and distribution, were obtained both from direct examinations and the literature, e.g., [11,12,14,15,34,35,36,37]. The list of cetrarioid core lichen species examined for this study is provided in Supplementary Table S3.

Taxon Sampling: A broad taxonomic sampling (all sequences retrieved from NCBI GenBank) was conducted to focus on the cetrarioid core group of Parmeliaceae, including 16 genera, 74 species, and 382 samples (Supplementary Table S1) [38,39]. Species of the genera *Dactylina*, *Esslingeriana*, and *Melanelia* were used as outgroups. These three genera belong to phylogenetically distant lineages of Parmeliaceae outside the cetrarioid core clade, which is suitable for rooting phylogenetic trees per previous published lichen systematic research [9,12,40]. Molecular analyses were based on a three-locus dataset, comprising the mitochondrial SSU (mtSSU), the internal transcribed spacer (ITS), and the nuclear ribosomal large subunit (nuLSU). Sequences for each locus were aligned using the MAFFT algorithm [41], and ambiguous regions of alignment were removed based on the GUIDANCE confidence score [42].

**Figure 1:**
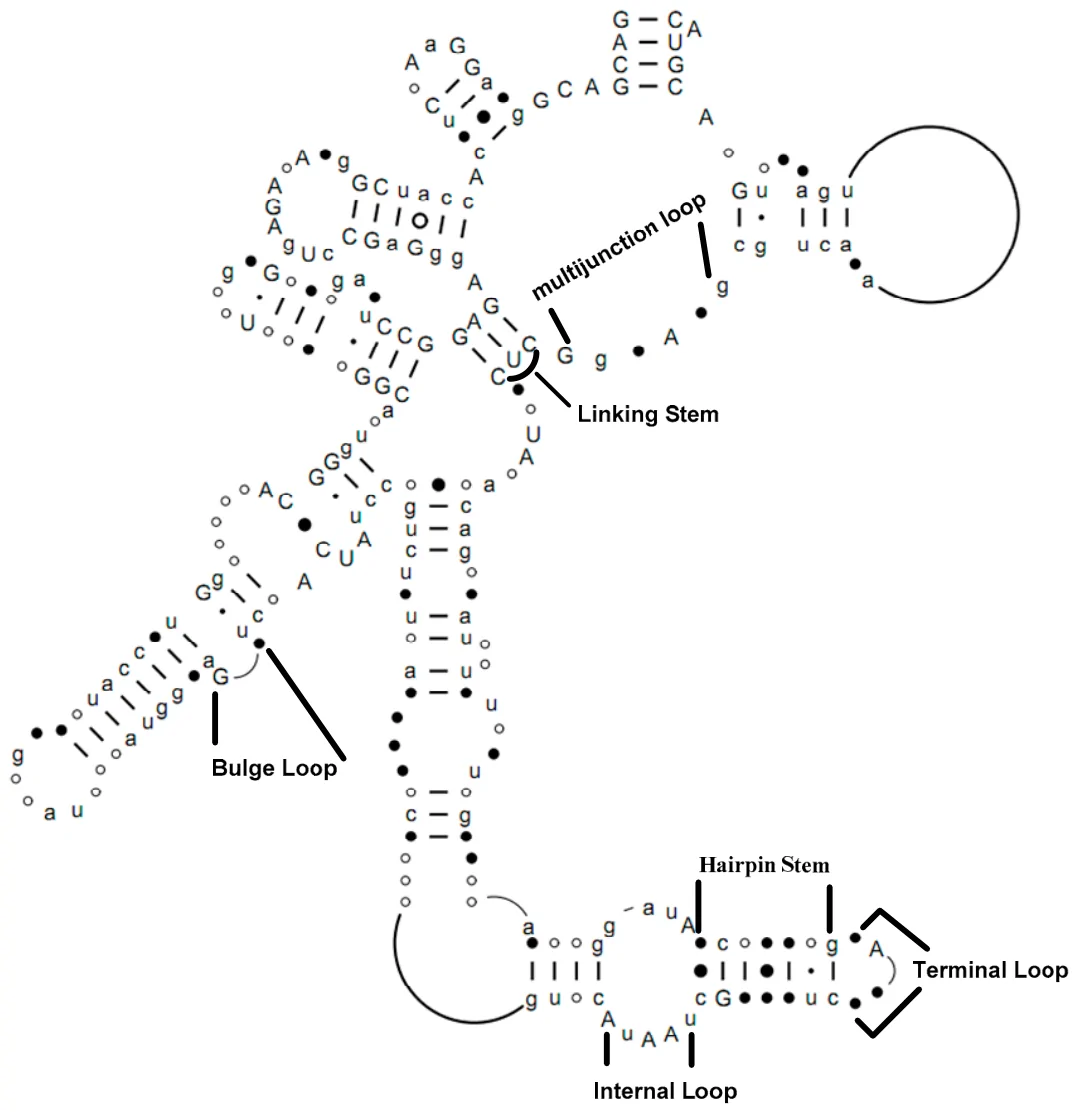
An example of the distribution of different types of data blocks in the secondary structure.

Data Partitioning and Model Selection: The secondary structure models J01393 and U53879, available in the Comparative RNA Web, were used as the reference models for the mtSSU and 5.8S-nuLSU regions of alignment in this study, respectively [43]. The alignment-based consensus structure models for ITS1 and ITS2 were predicted using RNAalifold from the ViennaRNA Package [44]. Each region (e.g., mtSSU, ITS1, ITS2, and 5.8S-nuLSU) in the alignment was divided into six data blocks based on structure features (Figure 1). These blocks consist of: BL (Bulge Loop—Bulges of unpaired bases on one strand of a stem); IL (Internal Loop—Unpaired regions linking neighboring helices); HL (Terminal Loop—Unpaired regions at the tip of a hairpin stem); ML (Multijunction Loop—A single stranded region into which more than two stems meet); HS (Hairpin Stem—Base pairs within closed helical regions); and LS (Linker Stem—Base pairs connecting adjacent helices). The remaining sites of alignment, which did not associate with specific features, were assigned to a separate block RM.

We defined a “data block” as a set of sites in an alignment, a “data subset” as a combination of one or more data blocks, and a partitioning scheme as a set of subsets containing all sites in the alignment. Upon availability of the data blocks, the corresponding primary partitioning scheme was generated, namely BL, IL, HL, ML, HS, LS, and RM. The primary scheme was then refined by merging data blocks with similar substitution rates into a single data subset using a greedy search algorithm called ModelFinder [45]. The optimal partitioning scheme and the best-fit substitution model for each data subset were obtained through subsequent searches.

Phylogenetic Inference: A partitioned maximum likelihood (ML) analysis was performed using IQ-Tree v.3.0 [46] with ultrafast bootstrap (1000 replicates) based on an optimal partitioning scheme comprising seven data subsets. Each subset was assigned a specific substitution model, as follows:

1. BL (mtSSU) + HL (mtSSU) + ML (mtSSU) + HS (mtSSU) + LS (mtSSU) + ML (5.8S-nuLSU): HKY+F+R3;
2. IL (mtSSU) + RM (mtSSU): HKY+F+G4;
3. BL (ITS1) + HL (5.8S-nuLSU): TN+F+R2;
4. HL (ITS1) + IL (ITS1) + ML (ITS1) + HS (ITS1) + BL (ITS2) + IL (5.8S-nuLSU): TIM2e+I+G4;
5. LS (ITS1) + HL (ITS2) + IL (ITS2) + ML (ITS2) + HS (ITS2): TIM2 + G4;
6. BL (5.8S-nuLSU) + HS (5.8S-nuLSU) + LS (5.8S-nuLSU): TIM + F + R2;
7. RM (5.8S-nuLSU): GTR + I + G4.

A partitioned Bayesian inference (BI) was conducted using the same partitioning scheme in MrBayes v.3.2 [47]. To sample across substitution models, the default settings (lset = mixed; statefreqpr = dirichlet (1,1,1,1)) were applied to each data partition. Markov chain Monte Carlo (MCMC) simulations were run for 8,000,000 generations; convergence was verified by ESS values >200. Trees were sampled every 1000 generations during MCMC runs. Bayesian posterior probabilities (PP) were estimated from a 50% majority-rule consensus of trees sampled after convergence to stationarity, following the discard of the initial 25% of samples as burn-in.

## 3. Results

**Figure 2:**
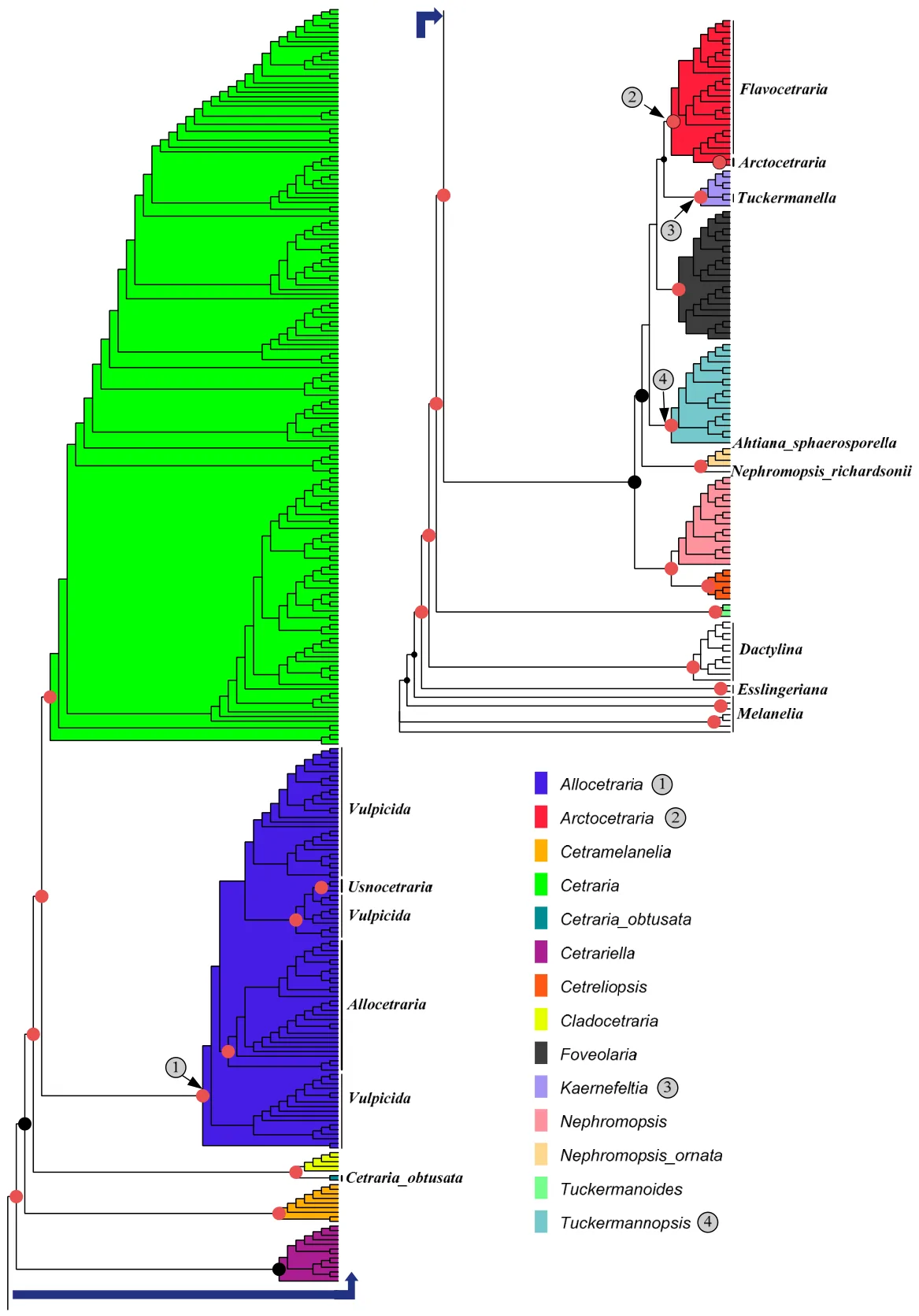
Maximum likelihood phylogenetic tree inferred from an optimal partitioned analysis based on the secondary structure features across three loci (ITS, nuLSU, and mtSSU). Node support is indicated by circles: red (BS ≥ 93% and PP ≥ 0.90), black (BS ≥ 93% or PP ≥ 0.90), and small black (90% ≤ BS < 93% and PP < 0.90).

The core ‘cetrarioid’ lichens constitute a significant informal group within the Parmeliaceae family, characterized primarily by their erect or sub-erect thalli and marginal apothecia or pycnidia. Previous phylogenetic analyses have demonstrated that the ‘cetrarioid core’ represents a distinct, monophyletic lineage, consistently split into two major lineages: the *Nephromopsis* and the *Cetraria* [5,9,20,48]. Our optimal partitioned phylogeny fully corroborates this topology based on extensive sampling and partitioned analysis incorporating rRNA secondary structure characteristics. All cetrarioid lichen taxa were found to form a strongly supported clade, with 94% bootstrap support (BS) and 1.00 posterior probabilities (PP). The two internal lineages also received robust nodal support (*Cetraria* lineage: 99% BS, 1.00 PP; *Nephromopsis* lineage: 98% BS, 0.90PP) (Figure 2).

The *Cetraria* lineage comprised 278 specimens (35 species, six genera: *Cetraria* s. str., *Vulpicida*, *Usnocetraria*, *Allocetraria*, *Cladocetraria*, and *Cetrariella*) and was subdivided into five well-supported clades. *Cetraria* s. str. (including type species *C. islandica*) was monophyletic (99% BS, 0.97 PP), containing *C. sepincola* (formerly orphaned). Previous phylogenetic assessments have demonstrated that *Cetraria* is not monophyletic within its traditional, broad circumscription, with several species falling outside the *Cetraria* s. str. clade. Specifically, *C. annae*, *C. obtusata*, and *C. sepincola* have been identified as orphaned taxa requiring taxonomic reassignment [5,18]. This study recovered the *Cetraria* s. str. clade, comprising 163 specimens representing 13 species except for *C. obtusata* and *C. endochrysea*.

The *Allocetraria–Usnocetraria–Vulpicida* complex formed a strongly supported clade (99% BS, 0.99 PP) sister to *Cetraria* s. str. Within this complex, *Allocetraria* (including *C. endochrysea*) was monophyletic (BS 98%; PP 1.00), which includes 28 specimens from seven species. *Usnocetraria oakesiana* formed a highly supported clade (BS 100%; PP 1.00) with three geographically distinct and sequence-divergent specimens. *Vulpicida* was non-monophyletic, comprising 56 specimens from six species, separating into several distinct clades.

*Cladocetraria*, established as a monotypic genus for *Cladocetraria minuscula*, was confirmed as monophyletic in this study, with five specimens forming a strongly supported clade (BS 100%; PP 1.00). This clade was associated with *Cetraria obtusata*. Together, they formed a well-supported clade (BS 100% BS; PP 1.00), being closely related to the *Cetraria–Allocetraria–Usnocetraria–Vulpicida* complex (BS 99%; PP 1.00).

*Cetrariella* was non-monophyletic: the type clade (*C. delisei*, *C. fastigiata*) was basal, which recovered as a well-supported monophyletic clade (BS 89%; PP 0.99), while *C. commixta* and *C. sorediella* formed a separate strongly supported clade (BS 100%; PP 1.00). This clade was closely related to the *Cetraria–Allocetraria–Usnocetraria–Vulpicida– Cladocetraria* complex (BS 98%).

The *Nephromopsis* lineage (BS 98%) (101 specimens, 28 species) included nine genera. Four were monophyletic: *Arctocetraria* (BS 96%; PP 0.91), *Tuckermanella* (BS 99%; PP 0.92), *Foveolaria* (BS 99%; PP 0.99), and *Cetreliopsis* (BS 98%; PP 0.91). *Flavocetraria* s. str. was non-monophyletic. *Kaernefeltia* was paraphyletic, with *Tuckermanella* nested within *Tuckermanopsis* (excluding *T. platyphylla*) forming a clade with *Ahtiana sphaerosporella*. *Nephromopsis* s. lat. was non-monophyletic; a core clade (*Nephromopsis* s. str.) received weak support.

The genus *Kaernefeltia* was found to be paraphyletic because *Tuckermanella* was nested within it. Together, they formed a highly supported clade (BS 97%; PP 0.99). Five *Tuckermannopsis* species, except *T. platyphylla*, formed a well-supported clade (BS 96%; PP 1.00), which also included *Ahtiana sphaerosporella*. The genus *Nephromopsis* was not resolved as monophyletic, with two species, *N. ornata* and *N. richardsonii*, clustering together to form a well-supported clade (BS 99%; PP 0.92) that separated from the core *Nephromopsis* clade. This core clade (corresponding to *Nephromopsis* sensu stricto and including the type species *N. stracheyi*) comprised 17 samples representing nine species, but received weak statistical support. The genus *Cetreliopsis*, represented by six samples from three species, was recovered as a well-supported monophyletic group sister to the core *Nephromopsis* clade (BS 98%; PP 0.91).

The *Nephromopsis* lineage was identified as a sister clade to the *Cetraria* lineage with strong support (BS 93%; PP 0.99), while a distinct third lineage, comprising three samples of *Tuckermanopsis platyphylla*, was recovered with strong support (100% BS, 1.00 PP), which is a sister to the *Cetraria*–*Nephromas* complex (BS 94%; PP 1.00).

## 4. Discussion

**Scheme 1:**
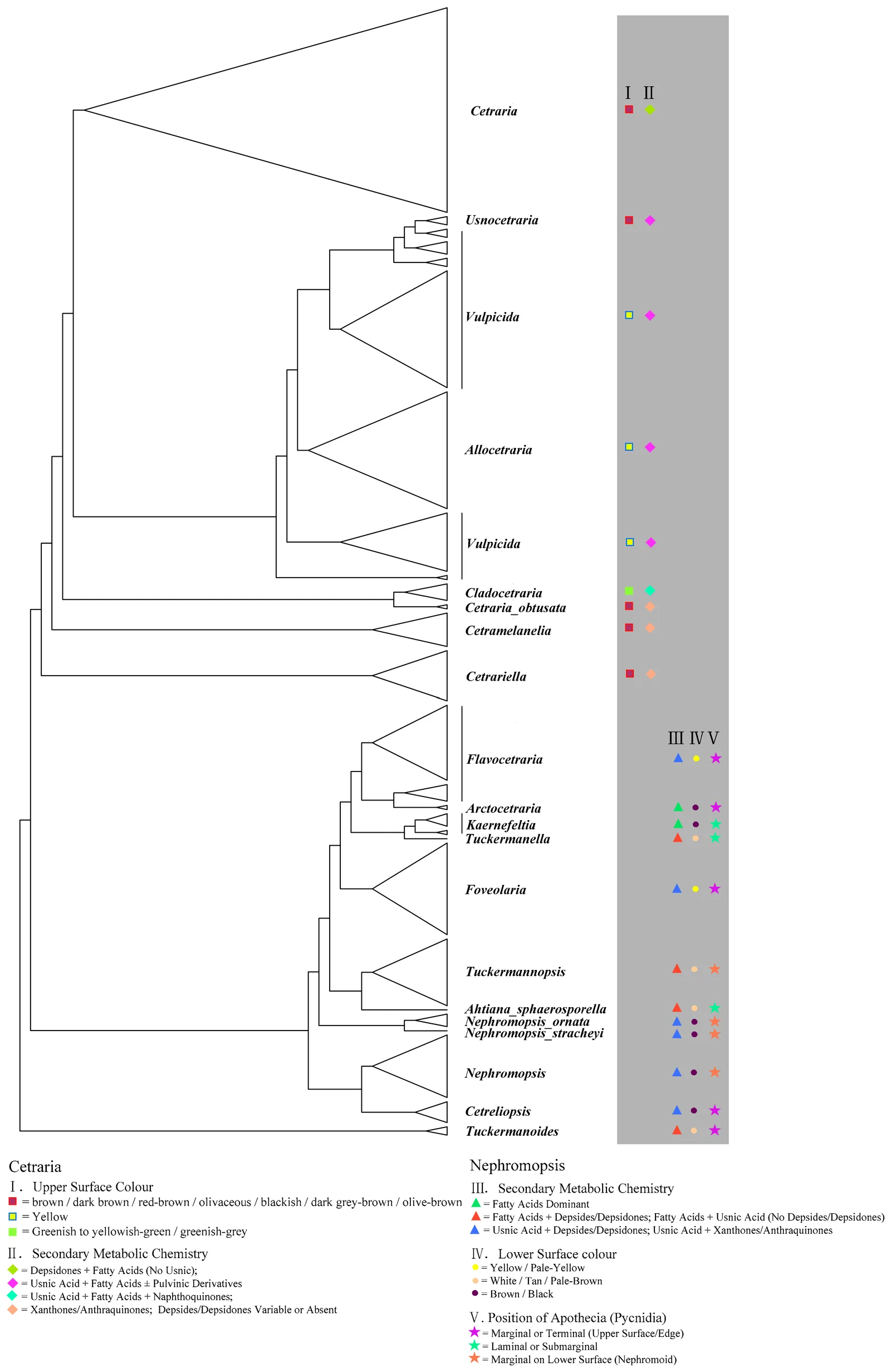
Mapping of phenotypic traits across the phylogenetic scheme. Colored symbols in the adjacent matrix indicate variations in morphological features. Character states are categorized into five main groups: upper surface color (I), secondary metabolic chemistry (II, III), lower surface color (IV), and the position of apothecia/pycnidia (V), with specific trait assignments detailed in the legend below.

Consistent with previous studies [9,10,12,19,40], conidial shape distinguished the two major lineages: filiform conidia in the *Cetraria* clade versus oblong/citriform/bifusiform conidia in the *Nephromopsis* clade [4,15]. However, conidial shape alone did not resolve intra-lineage relationships. Upper surface color showed a graded transition from dark (basal) to light (terminal) within the *Cetraria* lineage, with exceptions due to parallel evolution. Secondary chemistry profiles correlated broadly with phylogeny (Scheme 1). In the *Nephromopsis* lineage, apothecial position varied among taxa: *Nephromopsis* and *Tuckermannopsis* species typically had apothecia marginal on the lower surface; *Ahtiana*, *Tuckermanella*, and *Kaernefeltia* showed laminal or submarginal positions; while *Arctocetraria*, *Flavocetraria*, and *Foveolaria* had apothecia marginal on the upper surface.

Divakar et al. (2017) [9] first introduced the time-band delimitation method to unify generic ranking across Parmeliaceae, which provided an important quantitative reference for lichen taxonomic revision and promoted standardized molecular dating-based classification. Nevertheless, rigidly applying a single uniform divergence time threshold across all cetrarioid genera conflicts with palaeoclimatic and phenotypic evidence for this group [5]. Lücking (2019) questioned the rigid use of temporal phylogenetic criteria for genus delimitation [30]. Thell and Divakar (2022) advocated retaining a broad concept of *Cetraria*, noting that splitting this group would create numerous additional genera despite its already narrow generic boundaries [14,49]. This topic is discussed in detail below.

### 4.1. Morphological and Chemical Traits

Traditionally, genera were defined based on thallus morphology, ascus apex structure, chemical profiles, the location of apothecia/pycnidia, cortical anatomy, ecological preferences, and photobiont associations [11,12,13,15]. However, these traits have proven to be unreliable indicators of evolutionary relationships when considered individually [10]. While some genera, such as *Arctocetraria*, *Cetreliopsis*, *Kaernefeltia*, and *Tuckermanella*, are monophyletic, others, including *Cetraria*, *Flavocetraria*, and *Tuckermannopsis*, are polyphyletic [10,19]. In these cases, species exhibiting similar characteristics are found across genetically distant lineages. This indicates that these traits are highly homoplastic, arising independently through convergent evolution rather than being derived from a common ancestor, or they may be restricted to specific sub-lineages without clarifying deeper phylogenetic relationships [9,40]. In contrast, conidial shape showed some congruence with the major evolutionary clades [15,18].

This study supports the use of conidial shape as a phylogenetic marker, as fusiform and non-fusiform conidia are associated with two distinct and well-supported evolutionary lineages. However, this trait alone cannot fully account for the relationships within each lineage. Within the *Cetraria* lineage, for example, *Cladocetraria* and *Cetramelanelia* (new genus proposed below) both produce bacilliform conidia but are not closely related. Similarly, *Cetraria obtusata*, *Cetrariella*, and some other *Cetraria* species, all of which have bottle-shaped conidia, do not cluster together in the phylogenetic tree as expected. Within the *Nephromopsis* lineage, most species have bifusiform/dumbbell conidia with similar morphology, indicating that this feature is insufficient to explain their phylogenetic positions. These findings suggest that conidial shape provides only limited phylogenetic resolution. Additional morphological traits should therefore be examined.

#### 4.1.1. The Color of the Upper/Lower Surface

The color of the upper surface was a useful morphological trait that partially correlated with molecular phylogeny within the *Cetraria* lineage. Basal taxa (e.g., *Cetraria obtusata*, *Cetramelanelia*, *Cetrariella*) typically exhibit black or brown colors, which gradually lighten along the middle and terminal branches of the phylogenetic tree, becoming yellowish-green in the *Cladocetraria* clade and yellow in the *Vulpicida–Usnocetraria–Allocetraria* clade. This graded transition from dark to light is consistent with the hypothesis that species with similar upper-surface colors were generally closely related. However, the phylogenetic position of the genus *Cetraria* deviated from this expected trend (Scheme 1). Although some *Cetraria* species had brown/black upper surfaces, these were not closely related to basal taxa with similar colors. These exceptions suggested that, while upper surface color is generally a useful phylogenetic indicator within the *Cetraria* lineage, exceptions likely resulting from parallel evolution do occur. Sometimes, the color of the lower surface was also considered to distinguish the genera. Generally, lower surface coloration transitions from basal to terminal branches, shifting from dark pigmentation (black or brownish-black in *Cetreliopsis* and *Nephromopsis*) through lighter tones (white to pale brown in *Tuckermannopsis*, *Ahtiana sphaerosporella*, and *Tuckermanella*) to yellow or pale yellow in terminal taxa such as *Foveolaria* and *Flavocetraria cucullata*. A more comprehensive interpretation requires the integration of additional morphological traits, such as conidial shape. It should be noted that these observed correlations between phenotypic traits and phylogenetic position do not necessarily imply adaptive evolution; formal ancestral-state reconstruction is required to test these hypotheses.

#### 4.1.2. Secondary Metabolic Chemistry

Upper-surface color of lichen thallus was generally correlated with secondary chemistry profile. Four distinct chemical profiles were recognized within the *Cetraria* lineage, each corresponding to phylogenetic position and thallus pigmentation: (1) Xanthones (e.g., secalonic acid) or Depsides/Depsidones (e.g., fumarprotocetraric acid, norstictic acid, gyrophoric acid, hiascinic acid, α-collatolic acid and alectoronic acid) were dominant, while fatty acids and usnic acid were absent. This profile was limited to the basal groups *Cetraria obtusata*, *Cetramelanelia*, and *Cetrariella*, all of which displayed dark black-brown upper surfaces. (2) Usnic acid and fatty acids were accompanied by prominent naphthoquinones (i.e., usnic acid + fatty acids + naphthoquinones). This combination was represented by the genus *Cladocetraria*, which typically exhibits greenish to yellowish-green upper surfaces. (3) Usnic acid and fatty acids were present, occasionally with pulvinic acid derivatives, but naphthoquinones were absent (i.e., usnic acid + fatty acids ± pulvinic derivatives). This profile corresponds to the *Vulpicida–Usnocetraria–Allocetraria* branch, which is characterized by bright yellow upper surfaces. (4) Usnic acid was absent, and the chemistry comprised fatty acids and depsidones (i.e., fatty acids + depsidones). This profile was unique to the genus *Cetraria*, in which the color of the upper surface proved variable (Scheme 1).

Compared to the *Cetraria* lineage, the *Nephromopsis* clade represented a larger and more complex lineage, containing a greater number of morphologically diverse independent genera. For the *Nephromopsis* lineage, morphological characteristics such as conidial shape and upper-surface color do not reflect molecular phylogeny. However, some phylogenetic trends are observable in secondary chemistry profile, lower surface color, and apothecial position. Three secondary chemistry profiles were identified, ranging from the basal to the terminal branches. The first profile, represented by the basal genera *Cetreliopsis* and *Nephromopsis*, is characterized by the presence of usnic acid and the consistent absence of fatty acids. Chemical combinations include usnic acid with depsides/depsidones or usnic acid with xanthones/anthraquinones, such as usnic acid + depsides/depsidones and usnic acid + xanthones/anthraquinones. The remaining taxa, which form the middle and terminal branches, are all distinguished by the presence of fatty acids, corresponding to the second and third profiles. The second profile involves combinations of fatty acids with either usnic acid or phenolic compounds such as depsides and depsidones (e.g., fatty acids + depsides/depsidones in *Tuckermanella* and *Tuckermannopsis*; fatty acids + usnic acid in *Ahtiana sphaerosporella*). The third profile, found in terminal branches (e.g., *Arctocetraria* and *Kaernefeltia*), is characterized by fatty acids as the sole major compound (Scheme 1).

#### 4.1.3. The Position of Apothecia and/or Pycnidia

The position of apothecia also varies phylogenetically across three distinct types: (1) marginal on the lower surface; (2) laminal or submarginal; and (3) marginal or terminal on the upper surface. Basal taxa, such as *Nephromopsis* and *Tuckermannopsis*, typically have apothecia positioned marginally on the lower surface (type 1). Taxa in the middle branches, including *Ahtiana sphaerosporella*, *Tuckermanella* and *Kaernefeltia*, exhibit a transition towards laminal or submarginal positions (type 2). Finally, terminal taxa, such as *Arctocetraria* and *Flavocetraria cucullata*, have apothecia positioned along the margins of the upper surface (type 3).

Apothecial position shows a phylogenetic trend from marginal on the lower surface in basal taxa to marginal on the upper surface in terminal taxa. However, this trait exhibits homoplasy, as distantly related taxa (e.g., *Cetreliopsis* and *Flavocetraria*) share similar apothecial positions despite their phylogenetic distance, suggesting convergent evolution rather than shared ancestry. Formal ancestral-state reconstruction is needed to test these hypotheses in the future.

However, none of the three morphological features fully accounts for the phylogenetic placement of specific taxa within the *Nephromopsis* lineage. For example, *Flavocetraria cucullata* and *Foveolaria* have usnic acid and depsides/depsidones, yet they are phylogenetically distant from other taxa with the same chemical profile. Similarly, *Arctocetraria* and *Kaernefeltia* possess dark brown to black lower surfaces but do not cluster with other taxa of similar coloration. Additionally, the genus *Cetreliopsis* is distinguished from other basal taxa by its apothecia being located on the upper surface or margin (type 3), rather than in the typical type 1 position. These inconsistencies suggest that homoplasy arising from parallel or convergent evolution can lead to similar character states in distantly related taxa within the *Nephromopsis* lineage. While these traits (secondary chemistry, upper/lower surface coloration, and apothecial/pycnidia position) do not fully account for the phylogenetic positions of all taxa, they perform better than other traditional morphological features such as thallus growth form alone, ascus apex structure, or the presence/absence of isidia. Taxa with similar trait states tend to be related (Scheme 1). Accordingly, secondary chemistry, upper/lower surface coloration, and apothecial/pycnidia position are potentially useful as phylogenetic indicators here and warrant further detailed investigation.

### 4.2. Dual Temporal Band Hypothesis for Generic Delimitation

Based on the current phylogeny inferred from partitioned analyses of secondary-structure character signals, and incorporating the cetrarioid molecular chronograms of Divakar et al. [9,40] as well as Miocene palaeoclimatic evidence [50,51,52,53], this study proposes a dual-temporal-band hypothesis for generic delimitation in Parmeliaceae. The Large Temporal Band (29.0–35.0 Mya) is retained as the universal cutoff for most early-diverging genera originating from late-Oligocene cooling, whereas the complementary Small Temporal Band (14.0–18.0 Mya) is designated for lineages diversified during the Mid-Miocene climatic transition (~16 Mya). Morphologically and ecologically distinct taxa such as *Allocetraria* (including *Usnocetraria* and *Vulpicida*) and *Cetrariella*, whose crown ages fall within 14.0–18.0 Mya, should not be synonymized under *Cetraria* following the rigid single 29.45–32.55 Mya benchmark of Divakar et al. [9]. This dual framework reconciles discrepancies among molecular dating, palaeoenvironmental history, and conventional morphology-based taxonomy.

We emphasize that the temporal thresholds proposed here (29.0–35.0 Mya and 14.0–18.0 Mya) are not derived from new molecular dating analyses performed in this study. Instead, they are based on the integration of: (i) published molecular chronograms for Parmeliaceae [9,40], which estimated the crown diversification of cetrarioid core lineages at ca. 29–35 Ma (late Oligocene) and the radiation of several recently diverged genera at ca. 14–18 Ma (mid-Miocene), and (ii) well-documented palaeoclimatic transitions, specifically the late Oligocene cooling event [50] and the Mid-Miocene Climatic Optimum transition [51,52,53]. The boundaries were selected to encompass the confidence intervals of published divergence time estimates while aligning with major palaeoclimatic events known to have influenced lichen diversification [52,53].

The ca. 16.0 Mya interval corresponds to the late stage of the Mid-Miocene Climatic Optimum (MMCO, 17.0–14.0 Mya). This critical geological period was marked by a rapid global transition from extreme warmth to cooler and drier conditions. The cooling event triggered the initial expansion of the Antarctic ice sheet, expanded temperate–alpine habitats across the Northern Hemisphere, forest recession, and the massive formation of open habitats including rock surfaces, tree bark, and tundra biomes [50,51]. These environmental shifts greatly expanded available ecological niches for lichens and drove rapid lineage diversification of the cetrarioid core group. The crown diversification of the cetrarioid core lichens was dated to 18.0–15.0 Mya (peak at 16.0 Mya), tightly coupled with this MMCO climatic transition. Their adaptive traits, including foliose to fruticose thallus form, thick cortex, and specific lichen acid accumulation, evolved in response to cool, dry high-latitude/alpine niches emerging after 16 Mya. Climate-mediated niche partitioning and allopatric isolation together facilitated rapid generic radiation of the whole core group [49,52,53].

A rigid uniform generic cutoff of 29.45–32.55 Mya across all cetrarioid Parmeliaceae is evolutionarily and taxonomically flawed and cannot serve as a universal standard [30]. First, cetrarioid lichens experienced two successive independent generic radiations separated by more than 10 million years: primary stem diversification triggered by late-Oligocene global cooling (29.0–35.0 Mya), and secondary crown radiation driven by the Mid-Miocene climatic turnover around 16.0 Mya (14.0–18.0 Mya). Divergent palaeoclimatic triggers and niche filtering underpin these two cladogenetic pulses, so enforcing one identical temporal threshold artificially erases hierarchical differences between asynchronous evolutionary radiations, conflicting with fundamental evolutionary principles. As calibrated on the molecular chronograms of Divakar et al. [9,40], the questioned genera (*Allocetraria* including *Usneocetraria* and *Vulpicida*, *Cetrariella*, etc.) diversified at 14.0–18.0 Mya, over 10 My younger than the proposed 29.0–33.0 Mya benchmark, and attained independent niche specialization exclusively after mid-Miocene environmental shifts. Second, diagnostic disparities in ascus type, conidia shape, thallus architecture, cortical anatomy, and lichen secondary chemistry robustly contradict forced synonymization; these consistent apomorphic traits represent long-term ecological isolation and adaptive divergence, which cannot be neglected merely by a single molecular age parameter. Third, cross-class and cross-subphylum comparison across Ascomycota confirms heterogeneous generic divergence ages throughout the phylum: Sordariomycete genera mostly diversified at 15.0–20.0 Mya, while *Morchella* originated >90.0 Mya [54,55], e.g., *Neurospora* crown node divergence age ca 12.0 Mya and *Saccharomyces* crown node divergence age ca 17.0 Mya. The uniform time-bound generic delimitation would erase genuine evolutionary radiation events. Cross-phylum comparisons in Ascomycota show widely varying generic divergence ages, and a rigid uniform temporal cutoff has been criticized [30]. Therefore, our dual-band criterion is taxonomically more rational.

We acknowledge that these temporal thresholds are conceptual hypotheses derived from published data rather than empirically calibrated divergence times from this study. The proposed dual-band framework requires testing through: (i) expanded molecular dating analyses with additional fossil calibrations specific to cetrarioid lichens; (ii) ancestral-state reconstruction to test correlations between phenotypic traits and palaeoclimatic shifts; and (iii) evaluation across additional Parmeliaceae lineages to assess the generality of this approach.

### 4.3. Taxonomic Stability and Alternative Classifications

The taxonomic revisions proposed below represent a compromise between the two-genus scheme of Divakar et al. (2017) [9] and the traditional 14-genus classification [56,57]. We argue that our seven-genus scheme balances phylogenetic accuracy with nomenclatural stability for the following reasons.

First, all recognized genera are monophyletic with strong statistical support (BS ≥ 93% or PP ≥ 0.90), ensuring that each genus represents a natural evolutionary lineage. Second, we minimize nomenclatural changes by retaining well-established genera (*Cetraria* s. str., *Cladocetraria*, *Nephromopsis*) and only splitting demonstrably non-monophyletic groups. Third, type species remain in their original genera where possible, preserving nomenclatural stability. Fourth, each genus is diagnosed by clear, observable phenotypic characters, facilitating practical identification.

Compared to the two-genus scheme [9], our approach preserves morphologically and ecologically distinct lineages that would otherwise be obscured, maintaining information content that is useful for field identification and ecological studies. Compared to the traditional 14-genus scheme [56,57], our approach eliminates artificial genera that are not supported by molecular data, reducing confusion from non-monophyletic groups. While any taxonomic revision introduces some nomenclatural changes, we argue that recognizing non-monophyletic taxa creates greater long-term instability by obscuring evolutionary relationships and diagnostic characters [30].

### 4.4. Limitations and Future Perspectives

Our sampling covers most widespread temperate/boreal cetrarioid taxa, but rare North American endemic populations of the newly proposed genus *Tuckermanoides* remain insufficiently collected due to limited field investigation access. Further field sampling across North America will supplement missing molecular data to verify the generic independence of *Tuckermanoides*. In addition, genome-scale data may help clarify deep intergeneric ambiguous nodes in future follow-up research.

An integrated approach using data from morphology, chemistry, ecology, geography, and molecular phylogenetic analysis (based on multi-locus genes) has made it possible to make a more harmonious taxonomic treatment for the cetrarioid core lichens.

**Taxonomic Treatment** (Table A1)

Recent molecular studies have significantly advanced our understanding of cetrarioid lichens, confirming that the group’s core members form a monophyletic group [4,9,12,15,18,40]. However, the phylogenetic relationships within this clade remain unresolved, primarily due to weak statistical support at deeper nodes. Two taxonomic approaches have been proposed. One approach, based on divergence-time criteria, recommends merging several smaller genera into two larger ones (*Cetraria* and *Nephromopsis*) to reflect estimated splitting times. Traditional lichenologists, however, opposed this merger, arguing that such broad “megagenera” would obscure significant morphologically diagnostic characters. Instead, they advocated maintaining smaller, morphologically distinct and phylogenetically well-supported genera, resolving non-monophyly through targeted species reassignments rather than the loss of key diagnostic traits [5,9,30].

Our results partially supported the taxonomic revision of *Flavocetraria* proposed by Chesnokov et al. [5], who suggested dividing the genus, as originally circumscribed, into three monotypic genera: *Flavocetraria* s. str. (corresponding to *Flavocetraria* s. str., including the type species *F. cucullata*), *Cladocetraria* (containing the former *F. minuscula*), and *Foveolaria* (containing the former *F. nivalis*). However, *Flavocetraria* s. str. was found to be non-monophyletic. Examination of 24 specimens of *Flavocetraria cucullata* with interspecific sequence variation indicated that some samples deviated from the core *Flavocetraria* clade and clustered closely with *Arctocetraria* instead.

The results of the present study support a compromise between these positions. Accordingly, the following series of taxonomic revisions is proposed. Based on ITS + nrLSU + mtSSU ML phylogeny with strong nodal support, plus morphology, chemistry, ecology, and distribution, we propose a seven-genus scheme with well-supported, taxonomically stable, diagnostically clear, and fully consistent modern phylogenetic systematics for the cetraioid core lichen. This resolves long-standing polyphyly, corrects artificial generic limits, and aligns clades with phenotypic and ecological coherence.

***Cetraria***

Retention of the core *Cetraria* s. str. clade is justified. This lineage is morphologically, anatomically, and chemically uniform, and forms a strongly supported monophylum in the ML tree. No adjustment is needed.

***Allocetraria*** (including *Usnocetraria* and *Vulpicida*)

This expanded circumscription is well-supported. *Vulpicida* and *Usnocetraria* nest within *Allocetraria* with strong support, and share cortical anatomy, chemistry, and ecological preferences. Merging them creates a single, cohesive, monophyletic genus.

***Cladocetraria*** (including *Cetraria obtusata*)

Including *Cetraria obtusata* in *Cladocetraria* is phylogenetically sound and practical. The two form a strongly supported sister clade, share similar conidia and secondary chemistry, and are clearly separated from *Cetraria* s. str. and *Cetrariella*. This combination forms a natural, diagnosable unit.

***Cetramelanelia*** C. Y. Liu & S.Y. Guo, **gen. nov.** (Proposed below) (*Cetrariella commixta* + *C. sorediella*)

These two species form a highly supported, distinct clade clearly separated from *Cetrariella* s. str. They differ in lobe morphology, cortex structure, conidia shape, chemistry, and ecology from the remaining *Cetrariella* species. This split resolves the former polyphyletic *Cetrariella*.

***Cetrariella*** s. str. (*Cetariella delisei* + *C. fastigiata*)

Restricting *Cetrariella* to these two species is optimal. They form a strongly supported, morphologically and chemically homogeneous clade that corresponds to the original type concept of the genus and preserves nomenclatural stability.

***Nephromopsis*** (following Divakar et al. 2017 [9], including *Foveolaria* [5] and excluding *Tuckermannopsis platyphylla*)

*Foveolaria* nests securely within *Nephromopsis* with strong support, while *Tuckermannopsis platyphylla* is excluded because it represents a separate, well-supported evolutionary lineage. The group is ecologically and morphologically coherent.

***Tuckermanoides*** C.Y. Liu & S.Y. Guo **gen. nov.** (Proposed below) (*Tuckermannopsis platyphylla*)

*Tuckermannopsis platyphylla* forms a strongly supported, isolated clade distinct from both *Nephromopsis* and core *Tuckermannopsis*. It has diagnostic morphological and chemical features that justify generic status. Meanwhile, it is endemic to North America.

Remarks on some genera with new combinations and new synonyms.

***Allocetraria*** Kurok. & M.J. Lai, Bull. Natn. Sci. Mus., Tokyo, B 17: 60 (1991)

= *Usnocetraria* M.J. Lai & J.C. Wei, J. Natnl. Taiwan Mus. 60: 57 (2007)

= *Vulpicida* J.-E. Mattsson & M.J. Lai, Mycotaxon 46: 427 (1993)

Type: *Allocetraria stracheyi* (C. Bab.) Kurok. & M.J. Lai (1991)

Description: **Thallus** foliose to subfruticose or fruticose, erect, suberect or rarely adnate, forming rosettes or tufts. Lobes dichotomously or subdichotomously branched, clearly longer than broad, dorsiventral. Upper surface yellow, yellowish-green, olive-green, gray-green, brownish to dark brown, occasionally wrinkled; **soredia** occasionally present on lobe margins (in some species, e.g., former *Usnocetraria*); **isidia** and **lobulae** absent. **Pseudocyphellae** present, angular to sublinear, mainly restricted to the lower surface, rarely on both sides or absent. Lower surface pale to dark; **rhizines** sparse or absent. **Upper cortex** palisade plectenchymatous, composed of one tissue type or rarely two tissue types; medulla white to yellowish, or bright yellow (in former *Vulpicida*).

**Apothecia** rare or not observed, submarginal to laminal or lateral, sessile; thalline margin present, often incurved. **Asci** 8-spored, broadly clavate to narrowly clavate; tholus with an apical amyloid ring structure (K/I+), Lecanora-type. **Ascospores** colorless, aseptate, ellipsoid to subglobose or globose.

**Pycnidia** frequent to rare, laminal or borne on projections, often blackened, with a two-layered wall; ostiole blackened. **Conidia** colorless, not bifusiform, highly variable in shape: filiform, sublageniform, fusiform, citriform, bacillariform, or crescent-shaped.

**Chemistry:** Usnic acid consistently present in the cortex; atranorin absent. Medulla with fatty acids (lichesterinic, protolichesterinic, caperatic acids) and/or secalonic acids; pulvinic acid derivatives (pinastric and vulpinic acids) present in some species (former *Vulpicida*).

**Ecology:** Mainly corticolous or terricolous, often at high altitudes; several species endemic to the Himalayas.

Diagnosis: *Allocetraria* differs from *Cetraria* s. str. in having a palisade plectenchymatous upper cortex (vs. prosoplectenchymatous cortex), and in the absence of gyrophoric and hiascic acids. Furthermore, *Cetraria* s. str. typically produces lemon-shaped conidia and forms richly branched, olive-brown tufts with laminal apothecia, whereas *Allocetraria* exhibits diverse conidial shapes (filiform, bottle-shaped, crescent-shaped, etc.) and frequently lacks apothecia. Differs from *Cetrariella* s. str. in having angular to sublinear pseudocyphellae (vs. absent or not angular in *Cetrariella*), and in the absence of the C+ red medullary reaction and wine-bottle-shaped conidia. Differs from *Cetromelanelia* in consistently containing usnic acid in the cortex, and in the absence of β-orcinol depsidones including fumarprotocetraric and protocetraric acids, and in the absence of alectoronic and α-collatolic acids.

Multi-gene analyses combining ribosomal RNA and protein-coding sequences have consistently demonstrated a close relationship among the genera *Allocetraria*, *Usnocetraria*, *Vulpicida*, and *Cetraria* s. str. [9,48]. The monophyly of *Allocetraria* and *Cetraria* s. str. is well supported, while *Usnocetraria*, represented by only one species (*U. oakesiana*), has shown inconsistent phylogenetic positions across various studies [15,18]. In these studies, *U. oakesiana* was recovered on a separate branch, close to *Vulpicida* or some *Cetraria*. Similarly, the monophyly of *Vulpicida* remained unresolved, with some studies indicating paraphyly and others supporting its monophyly [18,20,58]. In the present study, three geographically distinct *U. oakesiana* specimens exhibiting genetic variation were analyzed and resolved as a highly supported monophyletic clade. Phylogenetic analysis of 56 specimens, representing all six *Vulpicida* species, reveals that the genus is not monophyletic, with its species instead distributed among several distinct clades. Notably, both *Usnocetraria* and *Allocetraria* are deeply embedded within the broader *Vulpicida* complex. Based on the close phylogenetic relationships and morphological similarities (e.g., upper surface color, chemical profile, and conidia shape) between the three genera, we propose merging *Vulpicida* and *Usnocetraria* into the genus *Allocetraria*.

Eight synonyms from *Cetraria* made by Wei (2020) [33] are accepted and six new combinations for *Allocetraria* are made here.

**Synonyms from** ***Cetraria*** **Made by Wei** [33]

*Cetraria corrugata* (R.F. Wang, X.L. Wei & J.C. Wei) Divakar, Crespo & Lumbsch, (MB 820138), Fungal Diversity 84:111 (2017), under **Allocetraria corrugata** R.F. Wang, X.L. Wei & J.C. Wei, Mycotaxon 130: 585 (2015).

*Cetraria endochrysea* (Lynge) Divakar, Crespo & Lumbsch, (MB 820139), Fungal Diversity 84:111 (2017), under **Allocetraria endochrysea** (Lynge) Kärnefelt & A. Thell, Nova Hedwigia 62: 507 (1996).

*Cetraria flavonigrescens* (A. Thell & Randlane) Divakar, Crespo & Lumbsch, (MB 820140), Fungal Diversity 84:111 (2017), under **Allocetraria flavonigrescens** A. Thell & Randlane, Flechten Follmann: 359 (1995)

*Cetraria isidiigera* (Kurok. & M.J. Lai) Divakar, Crespo & Lumbsch, (MB 820141), Fungal Diversity 84:111 (2017), under **Allocetraria isidiigera** Kurok. & M.J. Lai, Bull. Natn. Sci. Mus., Tokyo, B 17: 62 (1991).

*Cetraria laii* Divakar, Crespo & Lumbsch, (MB 820142), Fungal Diversity 84:111 (2017), under **Allocetraria stracheyi** (C. Bab.) Kurok. & M.J. Lai, Bull. Natn. Sci. Mus., Tokyo, B 17: 62 (1991)

*Cetraria sinensis* (X.Q. Gao) Divakar, Crespo & Lumbsch, (MB 820143), Fungal Diversity 84:111 (2017), under **Allocetraria sinensis** X.Q. Gao, Flechten Follmann: 365 (1995)

*Cetraria wangii* Divakar, Crespo & Lumbsch, (MB 820145), Fungal Diversity 84:111 (2017), under **Allocetraria yunnanensis** R.F. Wang, L.S. Wang & J.C. Wei, Lichenologist 47: 33 (2015)

*Cetraria weii* Divakar, Crespo & Lumbsch, (MB 820146), Fungal Diversity 84:111 (2017), under **Allocetraria capitata** R.F. Wang, L.S. Wang & J.C. Wei, Mycosystema 33: 21 (2014)

**New Combinations**

**Allocetraria canadensis** (Räsänen) C.Y. Liu & S.Y. Guo, **comb. nov.** (**FN573915**)

Basionym: *Cetraria juniperina* var. *canadensis* Räsänen, Ann. Missouri Bot. Gard. 20: 12 (1933). Type: Canada, Kamloops, Fish Lake; Kujula, 1931 (H, holotype).

≡ *Vulpicida canadensis* (Räsänen) J.-E. Mattsson & M.J. Lai, Mycotaxon 49: 427 (1993).

**Allocetraria juniperinus** (L.) C.Y. Liu & S.Y. Guo, **comb. nov.** (**FN573916**)

Basionym: *Lichen juniperinus* L., Spec. Plant.: 1147(1753) (nom. cons.). Type: Sweden, Härjedalen, Storsjö; Mattsson 2340, 1991 (LD, neotype; typ. cons.).

≡ *Vulpicida juniperinus* (L.) J.-E. Mattsson & M.J. Lai, Mycotaxon 49: 427(1993).

**Allocetraria pinastri** (Scop.) C.Y. Liu & S.Y. Guo, **comb. nov.** (**FN573917**)

Basionym: *Lichen pinastri* Scop., Flora Carniolica 2: 382 (1772). Type: Italy, Friuli, Prov. Udine; Nimis, 1993 (LD, neotype)

≡ *Vulpicida pinastri* (Scop.) J.E. Mattsson & M.J. Lai, Mycotaxon 49: 428 (1993).

**Allocetraria tilesii** (Ach.) C.Y. Liu & S.Y. Guo, **comb. nov.** (**FN573918**)

Basionym: *Cetraria tilesii* Ach., Syn. Meth. Lich.: 228 (1814). Type: (Russia), Kamtschatka; Tilesius (H-ACH 1519, lectotype; UPS, isotype)

≡ *Vulpicida tilesii* (Ach.) J.-E. Mattsson & M.J. Lai, Mycotaxon 49: 428 (1993).

**Allocetraria tubulosus** (Schaer.) C.Y. Liu & S.Y. Guo, **comb. nov.** (**FN573919**)

Basionym: *Cetraria juniperina* var. *tubulosa* Schaer. Lich. Helvet. Spicil.: 372 (1836). Type: (Switzerland), Mt. Gemmi; Guthmick 1853 (G, lectotype)

≡ *Vulpicida tubulosus* (Schaer.) J.-E. Mattsson & M.J. Lai, Mycotaxon 49: 428 (1993).

**Allocetraria viridis** (Schwein.) C.Y. Liu & S.Y. Guo, **comb. nov.** (**FN573920**)

Basionym: *Cetraria viridis* Schwein. in Halsey, Ann. Lyceum Nat. Hist. New York 1: 16(1824). Type: USA, New York, Long Island, Riverhead; Brodo 1094, 1960 (CANL 15631 neotype; COLO 179368, isotype)

≡ *Vulpicida viridis* (Schwein.) J.-E. Mattsson & M.J. Lai, Mycotaxon 49: 428(1993).

***Cladocetraria*** Chesnokov, Prokopiev & Konoreva, Pl. Syst. Evol. 309(no. 24): 10 (2023)

One new combination.

**Cladocetraria obtusata** (Schaer.) C.Y. Liu & S.Y. Guo, **comb. nov.** (**FN573923**)

Basionym: *Cetraria aculeata* Fr. d. *obtusata* Schaer., Lich. Helvet. Spicil. 4-5: 225. (1833). Type: (Switzerland), (Alps), Mt. Grimsel; Schaerer (G, lectotype)

≡ *Cetraria obtusata* (Schaer.) Van den Boom & Sipman, Lichenologist 26: 106 (1994).

***Nephromopsis*** Müll. Arg., Flora 74: 371 (1891)

One new synonym.

*Foveolaria nivalis* (L.) Chesnokov, Prokopiev, Konoreva & Davydov, (MB 845177), Pl. Syst. Evol. 309(no. 24): 15 (2023), **new syn.** under

**Nephromopsis nivalis** (L.) Divakar, A. Crespo & Lumbsch, Fungal Diversity 84: 113 (2017)

Two new genera with three new combinations.

***Cetramelanelia*** C.Y. Liu & S.Y. Guo, **gen. nov.**

Fungal Name: FN573911

Type: *Cetramelanelia commixta* (Nyl.) C.Y. Liu & S.Y. Guo

*Etymology*: The generic name comes from the combination of *Cetraria* and *Melanelia*, which have the main features of the two genera.

Description: Thallus foliose to subfruticose, 2–5 cm-wide, loosely attached to substrate. Upper surface dark brown to black, smooth to slightly wrinkled, often pruinose. Lower surface distinct black, with sparse to moderate rhizines. Upper cortex paraplectenchymatous, 5–6 layers, 15–40 μm-thick; lower cortex similar, 4–5 layers, 15–45 μm-thick. Apothecia marginal on lower surface, rare. Conidia citriform, 4–6 × 1–1.5 μm. Chemistry: alectoronic and/or α-colllatolic acids present, usnic acid absent.

Diagnosis: Differs from *Cetrariella* s. str. by citriiform conidia (vs. bottle-shaped or sublageniform, 5–7 × 1.5–2 μm), distinct cortical anatomy (thinner cortex with smaller cells), and saxicolous (vs. terricolous). Differs from *Melanelia* by marginal apothecia (vs. laminal), absence of usnic acid, and darker thallus pigmentation. Differs from *Cetraria* s. str. by absence of fumarprotocetraric acid and different conidial shape.

**Cetramelanelia commixta** (Nyl.) C.Y. Liu & S.Y. Guo, **comb. nov.** (**FN573921**)

Basionym: *Platysma commixtum* Nyl., Syn. meth. lich. (Parisiis) 1(2): 310 (1860). Type: Finland, Helsingfors; Nylander, 1860 (H, lectotype)

≡ *Cetrariella commixta* (Nyl.) A. Thell & Kärnefelt, in Thell, Feuerer, Kärnefelt, Myllys & Stenroos, Mycol. Progr. 3(4): 309 (2004).

≡ *Melanelia commixta* (Nyl.) A. Thell, Nova Hedwigia 60(3-4): 417 (1995).

≡ *Cetraria obtusata* (Schaer.) Van den Boom & Sipman, Lichenologist 26: 106 (1994).

≡ *Tuckermannopsis commixta* (Nyl.) W.A. Weber, Bryologist 95(4): 403 (1992).

≡ *Parmelia fahlunensis* var. *commixta* (Nyl.) Boistel, Nouv. Fl. Lich. (Paris) 2: 70 (1903).

**Cetramelanelia sorediella** (Lettau) C.Y. Liu & S.Y. Guo, **comb. nov.** (**FN573922**)

Basionym: *Cetraria commixta* f. *sorediella* Lettau, Hedwigia 60: 119 (1918). Type: Switzerland, Engadin; Lettau, 1912 (B 13052, lectotype)

≡ *Cetrariella sorediella* (Lettau) V.J. Rico & A. Thell, in Nelsen, Chavez, Sackett-Herman, Thell, Randlane, Divakar, Rico & Lumbsch, Lichenologist 43(6): 548 (2011).

≡ *Melanelia sorediella* (Lettau) V.J. Rico, van den Boom & Barrasa, Lichenologist 37(3): 205 (2005).

≡ *Melanelia commixta* var. *sorediella* (Lettau) Hafellner & Türk, Stapfia 76: 153 (2001).

≡ *Cetraria sorediella* (Lettau) V.J. Rico, Fourth International Mycological Congress Abstracts (Regensburg): 49 (1990).

≡ *Cetraria fahlunensis* var. *sorediella* (Lettau) Räsänen, Kuopion Luonnon Ystâvâin Yhdistyksen Julkaisuja, ser. B 2(no. 6): 21 (1952).

***Tuckermanoides*** C.Y. Liu & S.Y. Guo, **gen. nov.**

Fungal Name: FN573912

Type: *Tuckermanoides platyphyllus* (Tuck.) C.Y. Liu & S.Y. Guo

*Etymology*: The generic name is named in honor of Edward Tuckerman, a pioneering American lichenologist.

Description: Thallus foliose, 3–8 cm-wide, broadly lobed (5–15 mm wide), loosely attached, broader in general than the other species of the *Tuckermannopsis* (*Nephromopsis*). Upper surface brown, rough, prominently wrinkled-reticulate, often with isidium-like laminal and marginal tubercles (but not true isidia). Lower surface black-brown to brown, with sparse rhizines. Apothecia marginal on the lower surface, rare. Conidia bifusiform, 5–7 × 1–1.5 μm. Chemistry: usnic acid absent; fatty acids (including caperatic acid) present.

Diagnosis: Differs from *Nephromopsis* by its isolated phylogenetic position (sister to the ‘*Cetraria–Nephromopsis*’ complex), brown broad foliose thallus with wrinkled-reticulate upper surface, unique fatty acid profile, and endemic North American distribution. Differs from ‘*Tuckermannopsis*’ s. str. by absence of usnic acid, different thallus morphology, and phylogenetic position. Differs from *Cetraria* by bifusiform conidia (vs. bacilliform or bottle-shaped) and different chemistry.

**Tuckermanoides platyphyllus** (Tuck.) C.Y. Liu & S.Y. Guo, comb. nov. (**FN573924**)

Basionym: *Cetraria platyphylla* Tuck., Syn. N. Amer. Lich. (Boston) 1: 34 (1882). Type: Canada, British Columbia, Stewarts Lake; Macoun s.n. (FH, lectotype)

≡ *Tuckermannopsis platyphylla* (Tuck.) Gyeln., Annls Mus. natn. Hung., Pars bot. 28: 282 (1934).

≡ *Nephromopsis platyphylla* (Tuck.) Herre, Proc. Wash. Acad. Sci. 12(2): 210 (1910).

The detailed description with illustration of the two new genera (*Cetramelanelia* and *Tuckermanoides*) is not included here; it will be published elsewhere with more specimens examined.
**Key to the Genera of the Cetrarioid Core Group (Parmeliaceae)** **1.** Conidia bifusiform or dumbbell-shaped (5–7 × 1–1.5 μm) .................................................................................................. **2** **1.** Conidia bacilliform, bottle-shaped, or citriform ................................................................................................................... **3** **2.** Thallus broadly lobed (5–15 mm wide), upper surface prominently wrinkled-reticulate; North American endemic; fatty acids only, usnic acid absent ............................................................................................................. ***Tuckermanoides*** **2.** Thallus not as above, upper surface color and chemistry variable; distribution not restricted to North America; chemistry variable; conidia bifusiform or dumbbell-shaped ................................. ***Nephromopsis*** (incl. ***Foveolaria***) **3.** Conidia citriform (lemon-shaped), 4–6 × 1–1.5 μm; upper surface dark brown to black; alectoronic acid and/or α-collatolic acid present, usnic acid absent; saxicolous ........................................................................ ***Cetramelanelia*** **3.** Conidia bacilliform or bottle-shaped ................................................................................................................................... **4** **4.** Usnic acid present (upper surface yellow to yellowish-green) ........................................................................................ **5** **4.** Usnic acid absent ..................................................................................................................................................................... **6** **5.** Naphthoquinones present (vulpinic acid, pinastric acid); upper surface yellowish-green; terricolous ........................................................................................................................ ***Cladocetraria*** (incl. ***C. obtusata***) **5.** Naphthoquinones absent; pulvinic acid derivatives may be present; upper surface bright yellow; corticolous to saxicolous ...................................................................................................... ***Allocetraria*** (incl. ***Usnocetraria***, ***Vulpicida***) **6.** Conidia bottle-shaped to sublageniform, 5–7 × 1.5–2 μm; thallus small, foliose; terricolous ............ ***Cetrariella*** s. str. **6.** Conidia bacilliform or bottle-shaped; thallus foliose to subfruticose, erect; ecology variable; fatty acids + depsidones; upper surface color variable ........................................................................................................................ ***Cetraria*** s. str.


## 5. Conclusions

Using an optimal partitioning scheme based on the secondary structure of rRNA genes, we obtained a strongly supported phylogeny of the cetrarioid core group. Combined with phenotypic traits and divergence time estimates from previous studies [9,40], we hypothesize a dual-band temporal criterion (Large Band: 29.0–35.0 Mya; Small Band: 14.0–18.0 Mya) for generic delimitation, as an alternative to the rigid single cutoff of 29.45–32.55 Mya. Our taxonomic revisions include the confirmation of the resurrection of *Allocetraria*, synonymisation of *Foveolaria* under *Nephromopsis*, and the tentative proposal of two new genera: *Cetramelanelia* and *Tuckermanoides*.

## Data Availability

All the data used to develop this manuscript are available through Supplementary Materials.

## Supplementary Materials

The following supporting information can be downloaded at: https://www.mdpi.com/article/10.3390/jof12090654/s1; Table S1: Data information and NCBI GenBank accessions of all voucher specimens (samples) used in this study; Table S2: A comparison of main characters among seven genera of the cetrarioid core group; and Table S3: List of the cetrarioid core lichen species examined for this study. File S1: sequence alignment matrix; File S2: partition scheme; and File S3: original ML tree.

## Appendix A

**Table A1:** The 2026 checklist of some cetrarioid core lichens (28 species in *Allocetraria*, *Cetramelanelia*, *Cetrariella*, *Cladocetraria* and *Tuckermanoides*). The name of the type species of each genus is in bold; Year and Name ID for the new combinations are also in bold. For species in the genera *Cetraria* and *Nephromopsis*, see Thell et al. 2005 [56], Randlane et al. 2013 [57], Divakar et al. 2017 [9], and Thell & Divakar 2022 [48].

Species and Authors	Year	Name ID
*Allocetraria ambigua* (C. Bab.) Kurok. & M.J. Lai	1991	IF354436
*Allocetraria capitate* R.F. Wang, Li S. Wang & J.C. Wei	2014	IF570064
*Allocetraria corrugata* R.F. Wang, X.L. Wei & J.C. Wei	2015	IF570087
*Allocetraria denticulata* A. Thell & Randlane	1995	IF415699
*Allocetraria endochrysea* (Lynge) Kärnefelt & A. Thell	1996	IF415617
*Allocetraria flavonigrescens* A. Thell & Randlane	1995	IF415700
*Allocetraria globulans* (Nyl.) A. Thell & Randlane	1995	IF415701
*Allocetraria isidiigera* Kurok. & M.J. Lai	1991	IF354961
*Allocetraria madreporiformis* (Ach.) Kärnefelt & A. Thell	1996	IF415618
*Allocetraria oakesiana* (Tuck.) Randlane & A. Thell	1995	IF415702
*Allocetraria potaninii* (Oxner) Randlane & Saag	1992	IF358987
*Allocetraria sinensis* X.Q. Gao	1995	IF415703
***Allocetraria stracheyi*** (C. Bab.) Kurok. & M.J. Lai	1991	IF354437
*Allocetraria subteres* (Asahina) J.C. Wei	2020	IF821260
*Allocetraria yunnanensis* R.F. Wang, X.L. Wei & J.C. Wei	2015	IF809069
		
*Allocetraria canadensis* (Räsänen) C.Y. Liu & S.Y. Guo	**2026**	**FN573915**
*Allocetraria juniperinus* (L.) C.Y. Liu & S.Y. Guo	**2026**	**FN573916**
*Allocetraria pinastri* (Scop.) C.Y. Liu & S.Y. Guo	**2026**	**FN573917**
*Allocetraria tilesii* (Ach.) C.Y. Liu & S.Y. Guo	**2026**	**FN573918**
*Allocetraria tubulosus* (Schaer.) C.Y. Liu & S.Y. Guo	**2026**	**FN573919**
*Allocetraria viridis* (Schwein.) C.Y. Liu & S.Y. Guo	**2026**	**FN573920**
		
		
***Cetramelanelia commixta*** (Nyl.) C.Y. Liu & S.Y. Guo	**2026**	**FN573921**
*Cetramelanelia sorediella* (Lettau) C.Y. Liu & S.Y. Guo	**2026**	**FN573922**
		
		
***Cetrariella***** *****delisei*** (Bory ex Schaer.) Kärnefelt & A. Thell	1993	IF360707
*Cetrariella fastigiata* (Delise ex Nyl.) Kärnefelt & A. Thell	1993	IF360708
		
		
***Cladocetraria***** *****minuscula*** (Elenkin & Savicz) Chesnokov, Prokopiev & Konoreva	2023	IF835265
*Cladocetraria obtusata* (Schaer.) C.Y. Liu & S.Y. Guo	**2026**	**FN573923**
		
		
***Tuckermanoides platyphyllus*** (Tuck.) C.Y. Liu & S.Y. Guo	**2026**	**FN573924**
