# Supplementary material for: Optimal Partitioning for Molecular Phylogenetic Inference and Generic Delimitation in the Cetrarioid Core Group (Parmeliaceae, Ascomycota)
Source: J Fungi (Basel). 2026 Sep 1;12(9):654. doi: 10.3390/jof12090654 (PMC13608483; doi:10.3390/jof12090654)
Supplement: Supplementary file 1 [file jof-12-00654-s001.zip › TableS2&S3.pdf]

Table S2. A comparison of main characters among seven genera of the cetrarioid core lichens

**Characters**	***Cetraria* s. str.**	***Allocetraria* (incl. *Usnocetraria*, *Vulpicida*)**	***Cladocetraria* (incl. *C. obtusata*)**	***Cetrariella* s. str.**	***Cetramelanelia***	***Nephromopsis* (incl. *Foveolaria*)**	***Tuckermanoides***
**Thallus type**	Foliose to subfruticose	Foliose	Foliose to subfruticose	Foliose	Foliose to subfruticose	Foliose to subfruticose	Foliose
**Upper surface color**	Variable	Bright yellow	Yellowish-green	Dark brown to black	Dark brown to black	Variable	Brown
**Lower surface color**	Black	Black	Black	Black	Black	Black to dark brown	Black-brown to brown
**Rhizines**	Present	Present	Present	Present	Present	Present	Sparse
**Apothecial position**	Marginal on lower surface	Marginal on lower surface	Marginal on lower surface	Marginal on lower surface	Marginal on lower surface	Variable	Marginal on lower surface
**Conidial shape**	Bacilliform /bottle-shaped	Bacilliform	Bacilliform	Bottle-shaped to sublageniform	Citriform	Bifusiform /dumbbell-shaped	Bifusiform
**Major chemistry**	Fatty acids + depsidones	Usnic acid + fatty acids ± pulvinic derivatives	Usnic acid + fatty acids + naphthoquinones	Depsides /depsidones	Alectoronic /α-collatolic acids	Variable	Fatty acids only
**Usnic acid**	Absent	Present	Present	Absent	Absent	Variable	Absent
**Ecology**	Arctic-alpine Terricolous	Arctic-alpine Coticolous, Terricolous Some endemic to Himalaya	Arctic-alpine Terricolous	Terricolous	Saxicolous	Arctic-alpine Terricolous, Corticolous, rarely Saxicolous	North American endemic Corticolous, Ramicolus
**Crown age (Mya)**	~30–35	~14–18	~29–35	~14–18	~14–18	~29–35	~29–35

**Species number**	8(?)	21	2	2	2	61(?)	1

Table S3. List of the cetrarioid core lichen species examined for this study

**Allocetraria 12 species**
Allocetraria ambigua
Allocetraria capitata
Allocetraria corrugata
Allocetraria denticulata
Allocetraria endochrysea
Allocetraria flavonigrescens
Allocetraria globulans
Allocetraria isidiigera
Allocetraria madreporiformis
Allocetraria sinensis
Allocetraria stracheyi
Allocetraria yunnanensis
**Cetraria 8 species**
Cetraria ericetorum
Cetraria hepatizon
Cetraria islandica
Cetraria laevigata
Cetraria nigricans
Cetraria odontella
Cetraria sepincola
Cetraria xizangensis

**Cetrariella 1 species**
Cetrariella delisei
**Cetreliopsis 4 species**
Cetreliopsis asahinae
Cetreliopsis endoxanthoides
Cetreliopsis laeteflava
Cetreliopsis rhytidocarpa
**Flavocetraria 2 species**
Flavocetraria cucullata
Flavocetraria nivalis
**Nephromopsis 14 species**
Nephromopsis ahtii
Nephromopsis hengduanensis
Nephromopsis komarovii
Nephromopsis laii
Nephromopsis laureri
Nephromopsis melaloma
Nephromopsis morrisonicola
Nephromopsis nephromoides
Nephromopsis ornata
Nephromopsis pallescens
Nephromopsis stracheyi
Nephromopsis togashii
Nephromopsis weii
Nephromopsis yunnanensis
**Tuckermanopsis 3 species**
Tuckermanopsis americana
Tuckermanopsis ciliaris
Tuckermanopsis microphyllica

**Usnocetraria 1 species**
Usnocetraria oakesiana
**Vulpicida 3 species**
Vulpicida juniperinus
Vulpicida pinastri
Vulpicida tilesii
